# Remote ischemic conditioning in active ulcerative colitis: An explorative randomized clinical trial

**DOI:** 10.1038/s41598-020-65692-9

**Published:** 2020-06-12

**Authors:** Line E. Godskesen, Thomas R. Lassen, Nichlas R. Jespersen, Majken Siersbæk, Yan Yan, Michael M. Nielsen, Sara K. Tjønnfjord, Lars Grøntved, Gunvor Madsen, Jørgen Kjems, Hans E. Bøtker, Michael R. Schmidt, Aleksander Krag, Jens Kjeldsen

**Affiliations:** 1 Department of Medical Gastroenterology, Odense University Hospital, Odense, Denmark; 2 Department of Cardiology, Aarhus University Hospital, Aarhus, Denmark; 3 Department of Biochemistry and Molecular Biology, University of Southern Denmark, Odense, Denmark; 4 Interdisciplinary Nanoscience Center (iNANO), Aarhus University, Aarhus, Denmark; 5 Department of Pathology, Odense University Hospital, Odense, Denmark; 6 Internal Medicine & Emergency Department, Odense University Hospital - Svendborg Sygehus, Svendborg, Denmark; 7 OPEN - Odense Patient data Explorative Network, Department of Clinical Research, University of Southern Denmark, Odense, Denmark; 8 Department of Clinical Research, University of Southern Denmark, Odense, Denmark; 9 Department of Molecular Biology and Genetics, Aarhus University, Aarhus, Denmark

**Keywords:** Ulcerative colitis, Randomized controlled trials

## Abstract

Remote ischemic conditioning (RIC) by repetitive brief periods of limb ischemia and reperfusion renders organs more resistant to ischemic injury. The protection is partly through down-regulation of the inflammatory response. Our aim was to investigate the clinical and anti-inflammatory effects of RIC in patients with active ulcerative colitis (UC). We included 22 patients with active UC in this explorative, randomized, sham-controlled clinical trial. The patients were randomly assigned 1:1 to RIC (induced in the arm through four cycles of 5-min inflation and 5-min deflation of a blood-pressure cuff) or sham (incomplete inflation of the blood-pressure cuff) once daily for 10 days. Outcome variables were measured at baseline and on day 11. When compared with sham, RIC did not affect inflammation in the UC patients measured by fecal calprotectin, plasma C-reactive protein, Mayo Score, Mayo Endoscopic Subscore, Nancy Histological Index or inflammatory cytokines involved in UC and RIC. The mRNA and miRNA expression profiles in the UC patients were measured by RNA sequencing and multiplexed hybridization, respectively, but were not significantly affected by RIC. We used the Langendorff heart model to assess activation of the organ protective mechanism induced by RIC, but could not confirm activation of the organ protective mechanism in the UC patients.

## Introduction

In spite of an increasing number of pharmacological treatment options to ulcerative colitis (UC) patients, a large number of UC patients experience insufficient disease control and a relapsing course of disease^1^. There is no known single cause for UC, but the general consensus is that in genetically susceptible individuals, environmental factors, and the composition of the gut microbiota can cause an aberrant activation of the innate and adaptive immune system leading to inflammation in the large intestine^2^.

The pathogenesis of UC is multifaceted including defective mucosal and epithelial barriers in the colon allowing microbiota to enter the intestinal lamina propria, activation and accumulation of neutrophils in the intestinal wall, up-regulated and more responsive pattern recognition receptors stimulating the adaptive immune system to increase homing of T and B cells^3^. Furthermore, dysregulated T cells enhance the inflammation by secretion of additional pro-inflammatory mediators such as tumor necrosis factor alpha (TNF-α), interferon gamma (IFN-γ), interleukin (IL)-1β, IL-6, IL-13, IL-17 and IL-23^4^. Finally, the regulatory T cells and their production of anti-inflammatory mediators like IL-10 fail to resolve the intestinal inflammation^5^.

Existing treatments are aimed at inhibiting different parts of the inflammatory response, and their treatment targets are to ameliorate the inflammation during active disease and subsequently maintain remission^6–8^.

Remote ischemic conditioning (RIC) by brief periods of non-lethal ischemia and reperfusion in a limb has been extensively studied for the last 3 decades, and although protection of the heart has seen most attention, the protective properties of RIC have also been demonstrated in other organs including the brain, liver, kidney and lungs^9,10^. The signal induced by ischemia in the limb is carried to the remote organ by neural and humoral pathways and by modification of the systemic inflammatory response^9–11^. RIC primarily reduces the ischemia/reperfusion injury (I/R injury), which is paradoxically induced when ischemic tissue is reperfused^12–14^. The inflammatory response induced by I/R injury resembles to some extent the inflammatory response in UC^15^. Studies suggest that RIC reduces activation of neutrophils after ischemic preconditioning^16^ and down-regulates genes involved in pro-inflammatory pathways including leukocyte activation, innate immunity, cell adhesion, and intracellular signaling^17^. RIC decreased neutrophil adhesion and had trend of decreased apoptosis in healthy volunteers undergoing treatment for 10 consecutive days. On the contrary, the same study demonstrated increased secretion of TNF-α, IL-10, IL-6, and IL-1 by neutrophils on day 10^18^. In animal models RIC reduced transcription factors NF-κB, TNF-α, IL-1β, and intercellular adhesion molecule 1 (ICAM-1) in intestinal I/R injury^19,20^, as well as TNF-α, IL-1β, IL-6, and IL-10 in mice with lipopolysaccharide induced sepsis^21^. These secondary effects are overall desirable in UC patients with active disease.

The neural pathway is known to involve the peripheral sensory nerves at the site where RIC is applied as well as the neuronal transfer to the target organ. This pathway presumably involves signaling from the affected peripheral sensory nerves to the spinal cord and via autonomic centers activate the vagus nerve^22^. The vagus nerve then activates the target organ by releasing acetylcholine which act on muscarinic receptors either directly in the target organ and/or indirectly in a non-target organ such as the spleen (the vago-splenic-axis) which releases humoral factor(s) that can act on the target organ^23^. Generally, the vagus nerve is also involved in immune regulation^24^. The anti-inflammatory effect of the vagus nerve is partly mediated through the hypothalamic-pituitary-adrenal axis (HPA axis) and partly through the cholinergic-anti-inflammatory-pathway^25,26^. Peripheral activation of the afferent vagus fibers transmit the neural signal to central nervous system and here activates the hypothalamic-pituitary-adrenal axis and hereby stimulates glucocorticoid secretion. Efferent vagal fibers release acetylcholine that binds to the α7 nicotinic acetylcholine receptor on macrophages and hereby attenuate the release of pro-inflammatory cytokines (i.e. TNFα and IL-6) from the macrophages.

I/R injury is not a part of the pathophysiology in UC, however, it is relevant to test RIC in the setting of a chronic inflammatory disease because of the suggested anti-inflammatory effects. Furthermore, RIC has a low side-effect rate and low economic cost.

The aim of this study was to investigate the effect of RIC on inflammation in patients with active UC. We examined if RIC attenuates inflammation measured by clinical, endoscopic and histological disease activity scores, as well as by serological and fecal surrogate markers of inflammation. We examined if RIC altered the expression of mRNA and miRNA. We examined how RIC was tolerated and accepted by UC patients. Furthermore, we explored whether the cardioprotective mechanism was activated by RIC in UC patients.

## Method

We conducted an explorative, randomized, single-blinded, sham-controlled clinical trial at the outpatient clinic at The Department of Medical Gastroenterology, Odense University Hospital, Denmark from June 2015 to February 2018.

### Patients

Eligible patients were 18 years or older, diagnosed with UC according to ECCO guidelines^27^ at least 6 months prior to inclusion and had moderate disease activity according to a Mayo Score ≥ 6 and an endoscopic Mayo Score > 1 at time of inclusion^6^.

Exclusion criteria were pancolitis or acute severe UC requiring immediate treatment; need for admission due to active ulcerative colitis; systemic symptoms (abdominal pain, fever > 38 degrees, weight loss exceeding 3 kilograms); anemia (hemoglobin <8.3 mmol/l for males and <7.3 mmol/l for females); stoma or pouch; bowel resection (except appendectomy); other known bowel condition than UC (coeliac disease, irritable bowel syndrome or constipation); diabetes; regular intake of acetylsalicylic acid or NSAIDs; colon cancer; dysplasia or adenomatous polyps in the colon during the recent 5 years; poor general condition; food poisoning within the last three months; medical treatment with cyclosporine at inclusion; changes in treatment with 5-aminosalicylic acid within two weeks prior to inclusion; changes in treatment with azathioprine, 6-mercaptopurine or methotrexate within 12 weeks prior to inclusion; changes in biological treatment within 12 weeks prior to inclusion; pregnancy at the time of inclusion or planned pregnancy during the study period; antibiotic treatment within two weeks prior to inclusion; failing to understand the written information. Finally, we excluded patients with any medical or surgical condition that excluded the use of RIC (systolic blood pressure of more than 180 mmHg, osteoporosis, the need of hemodialysis, hemophilia, anticoagulant or antiplatelet therapy, abnormal blood flow or nerve supply to the arms, peripheral neuropathy or pre-existing traumatic injury to the arms).

### Study design

The patients were randomized 1:1 to 10 days of remote ischemic conditioning (intervention group) or sham (control group). RIC was induced by intermittent upper arm ischemia produced by 4 cycles of alternating 5-minute inflation to 200 mmHg and 5-minute deflation of an upper arm blood pressure cuff. One intervention of 4 cycles was performed daily for 10 days. Participants allocated to the sham group underwent the same daily use of a blood pressure cuff, but the cuff pressure was only 20 mmHg at inflation. The blood pressure cuffs used in the study were autoRIC Device (CellAegis Devices Inc., 6711 Mississauga Rd, Suite 109, Mississauga, Ontario, Canada).

The randomization was in blocks of 6 and was generated by the data manager using Sealed Envelope^28^ and incorporated into the study database created in REDCap. The attending physician did the allocation in a room separate from the patient. On day 11 the study intervention was terminated, the patients unblinded and medical treatment was adjusted if needed. The patients were followed in the study for 30 days.

During the intervention period the patients were contacted daily by telephone, and data for the partial Mayo Score, use of autoRIC Device and side effects were collected. Standard blood samples for hemoglobin, plasma C-reactive protein (p-CRP) and leucocytes were collected on days 4 and 7 to evaluate the need for pre-term exclusion.

During the intervention period all patients were contacted daily by telephone and data for the partial Mayo Score, use of autoRIC Device and side effects were collected. Standard blood samples for hemoglobin, C-reactive protein (CRP) and leucocytes were collected on day 4 and 7 to evaluate the need for pre-term exclusion.

Due to a low inclusion rate the study site was expanded to include the outpatient clinic at Internal Medicine - Gastroenterology, Odense University Hospital - Svendborg Sygehus, Denmark from January 2017 February 2018 and the outpatient clinics at Department of Gastroenterology, Sydvestjysk Sygehus, Denmark and Department of Internal Medicine, Section of Gastroenterology, Sygehus Lillebælt - Vejle Sygehus, Denmark from April 2017 to February 2018. The patients were given an economic compensation of 1,500 Danish kroner to cover lost income, transportation costs, pain and suffering. Furthermore, the lower limit for Mayo Score at inclusion was changed from Mayo Score > 6 to Mayo Score ≥ 6.

### Outcome measures

The primary clinical outcome was a difference in fecal calprotectin (FC) from day 1 to day 11. Secondary endpoints were changes in Mayo Score, Mayo Endoscopic Score, Nancy histological index for UC^29^, Geboes histological subscore Grade 2b (Lamina propria neutrophils) and Grade 3 (Neutrophils in epithelium)^30^, and p-CRP, as well as a number of patients achieving remission (Mayo Score ≤ 2), changes in the levels of inflammatory cytokines, and alternations in the mRNA and miRNA expression profiles. Feasibility and tolerability were assessed by the number of side effects occurring and compliance to use the autoRIC Device.

### Cytokines

We measured the plasma levels of IL-1β, IL-2, IL-4, IL-6, IL-10, IL-12/IL-23p40, IL- 12p70, IL-13, IL-17a, IL-17f, IL-18, IL-21, IL-22, IL-23, IL-27, IL-33, matrix metalloproteinase 1 (MMP-1), MMP-2, MMP-3, MMP-7, MMP-8, MMP-9, MMP-10, tissue inhibitors of metalloproteinase 1 (TIMP-1), ICAM-1, IFN-γ and TNF-α. Plasma levels were measured using V-PLEX, U-PLEX and R-PLEX Immunoassay and a SECTOR S 600 (Meso Scale Discovery). The assay uses sandwich immunoassay and electrochemiluminescent-labeled antibodies to quantify the amount of cytokine in a sample. Depending on availability the assays were run as single or multiplex.

The cytokines were measured at 3 time points; pre-intervention at day 1, 15 minutes post intervention at day 1 and at day 11. The plasma samples were diluted and run according to manufacturer’s protocol. Calibrator standards and plasma samples were run in duplicates. Based on 8 standard concentrations, a standard curve for each cytokine and for each plate was fitted to a 4-parameter logistic curve. Detection range was defined by the average concentration of the zero standard, plus 2.5 times the standard deviation of the zero concentration as the lower limit of detection, and the concentration of the top of the standard curve as the upper limit of detection. The fit-curve range was defined by the estimated coefficients of the fitted curve.

We considered measurements valid when both assessments of a sample were within the range of detection, within the fit-curve range, and the coefficient of variation (CV) was <20%. Assays with more than 60% (35) valid samples per cytokine were selected for further statistical analyses. The samples were analyzed at Nordic Bioscience A/S (Herlev Hovedgade 205–207, 2730 Herlev, Denmark).

### mRNA isolation and sequencing from rectal biopsies

Rectal biopsies from the RIC group at day 1 (n = 11) and day 11 (n = 11), from the sham group at day 11 (n = 10), and from HC (n = 6) were used for the RNA sequencing (RNA-seq).

The RNA isolation and sequencing was carried out as described elsewhere^31,32^. In short, the biopsies were homogenized with Ultra-Turrax, and total RNA was extracted and purified from the samples using Isol-RNA Lysis Reagent (Thermo Fisher) and EconoSpin columns (Epoc Life) according to manufacturer’s protocols. RNA quality was assessed using the Fragment Analyzer (AATI).

cDNA libraries were prepared using 1 μg of RNA that was depleted for ribosomal RNA using Oligo d(T)_25_ Magnetic Beads. Library preparation was performed using the NEBNext RNA library prep kit for Illumina. Library quality was assessed using the Fragment Analyzer followed by library quantification (Illumina library quantification kit). Sequencing was carried out on a HiSeq. 1500 platform (Illumina).

We used the DAVID Bioinformatics Resources 6.8 for the GO term enrichment analysis (Laboratory of Human Retrovirology and Immunoinformatics (LHRI), Leidos Biomedical Research, Inc, Maryland)^33,34^.

### miRNA isolation and profiling from plasma samples

For miRNA profiling we used plasma samples from the RIC and sham groups at day 1 before RIC/sham intervention (n = 22); at day 1 15 minutes post the first RIC/sham intervention (n = 14) and at day 11 (n = 22), as well as plasma samples from HC (n = 10). We used the NanoString nCounter Human miRNA Expression Assay Kit (http://www.nanostring.com, NanoString Technologies, Seattle, Washington, USA) to profile 798 human miRNAs.

Hemolysis was measured as the absorbance at 414 nm, A_414_, using the NanoDrop spectrophotometer. Absorbance exceeding 0.2 indicates hemolysis and the sample should not be included (NanoDrop Technologies, Thermo Fisher Scientific, Waltham, Massachusetts, USA). We used the Plasma/Serum Circulating and Exosomal RNA Purification Mini Kit (Slurry Format, Norgen Biotek, Thorold, Canada) to isolate miRNA from 0.5 ml of EDTA-plasma. The synthetic miRNA oligonucleotides (spike-in oligos, 1000 attomoles/spike-in) ath-miR-159a and cel-miR-248 were added into the samples 5 minutes after adding lysis buffer (Operon, Inc.). miRNA was eluted in 100ul RNase-free water and precipitated by adding 1ul glycol-blue, 10ul 3 M NaOAc, 250ul ethanol and incubating at −20 °C overnight. miRNA was pelleted by centrifugation (16000 g, 10 minutes, 4 °C) and was washed using 1 ml 80% ethanol. Finally, miRNA pellet was resuspended in 8ul RNase-free water.

Three microliters of purified miRNAs were analyzed using the nCounter (NanoString Technologies, Seattle, Washington, USA) with the human miRNA assay kit (NanoString Technologies, Seattle, Washington, USA). Data was extracted and analyzed using the nSolver Analysis Software V 4.0. We removed miRNAs with expression below background level in 90% or more of the samples. All procedures were done according to the manufacturer’s instructions.

### The Langendorff heart model

Activation of the protective mechanism of RIC in human study subject can be evaluated in rat hearts in the Langendorff heart model^35,36^.

Sixty ml of whole blood were collected from the patients at 3 time points; pre-intervention at day 1, 15 minutes post intervention at day 1, and at day 11. Plasma was obtained from the whole blood within 10 minutes of blood collection by centrifuging at 2900 rpm for 20 minutes at 4 °C. The plasma was stored at −80 °C.

For the Langendorff experiment we used 39 Sprauge-Dawley rats (260–340 g) at 8 weeks of age. The animals where kept at 23 °C, 12/12 hours light and dark cycle. They had free access to food and water and were transported to experimental area the day before the experiment to reduce stress.

The Langendorff heart model was prepared as previously described^35,37^. In brief, general anesthesia was induced in the rats by subcutaneous injection with a mixture of fentanyl, fluanisone (hypnorm, VetaPharma, Leeds, UK) and midazolam (Hameln Pharma Plus, Hameln, Germany) 1.8 ml/kg body weight. A tracheotomy was made and the rat was ventilated by a rodent ventilator (Ugo Basile, Comerio, Italy). 500 IU/kg heparin (LEO Pharma, Ballerup, Denmark) was administered in the femoral vein, then a contentiously buffer-perfused metal cannula was placed in the aorta and the heart was rapidly excised and mounted in the Langendorff perfusion apparatus (IH-SR type 844/1; HSE, March-Hugstetten, Germany). In the Langendorff apparatus the heart was perfused with modified Krebs-Henseleit (KH) buffer mmol/l: NaCl 118.5, KCl 4.7, NaHCO_3_ 25.0, glucose monohydrate 11.0, MgSO_4_·7H_2_O 1.2, CaCl_2_ 2.4 and KH_2_PO_4_ 1.2) saturated with 95% O_2_ and 5% CO_2_ and preheated to 37 °C. A latex balloon was placed in the left ventricle and inflated with fluid to simulate preload and measure left ventricular developed pressure.

At the end of the Langendorff protocol, the hearts were frozen at −80 °C before being sliced into 6, 15 mm thick slices using a rat heart slicer matrix (Zivic Instruments, Pittsburgh, PA, USA) and stained with 1% 2,3,5-triphenyl tetrazolium chloride solution (Sigma-Aldrich, St. Louis, MO, USA) (in Sorensen’s phosphate buffer, pH 7.4, (Ampliqon, Odense, Denmark)) to visualize the infarct. Immediately after staining the hearts were stored in 4% formalin (VWR International, Leuven, Belgium) for 24 hours. The heart slices were weighed and scanned (Epson Perfection V600 Photo scanner, Epson, Nagano, Japan) and all images were assessed digitally to measure infarct size and area-at-risk using ImageJ software (NIH, Bethesda, Maryland, USA). Finally, the weight-adjusted infarct size/area-at-risk ratio was calculated. The experiment was done at The Department of Cardiology - Research Unit, Aarhus University Hospital - Aarhus, Denmark.

The isolated hearts were studied in 12 patients; 6 patients from the RIC group and 6 patients from the sham group. The Langendorff analyses were run in 2 setups, stratified over the groups. In the first setup the plasma from each patient from the three different time points was added to the perfusate with a syringe pump (1:6 dilution volume ratio of coronary flow) prior to the heat exchanger. Infusion flow of plasma was adjusted to the flow of each heart and thus varies from heart to heart. The statistical analysis of this data revealed large variance within each group. To enhance, control and align the amount of plasma perfused in each heart and hereby try to stabilize the model, the following changes were made in the second setup: The infusion flow was adjusted to 1:6 dilution volume ratio of total setup-flow, leading to less variation between hearts. Perfusion time was reduced to 5 minutes due to limited amount of plasma and there was no washout. Preliminary experiments were done in 3 healthy young males to ensure that the RIC-induced cardioprotection could be transferred to and detected in the Langendorff setup.

### Sample size

Since this is the first study of RIC in patients with inflammatory bowel disease, the effect of the intervention on patients was not known. The study is therefore exploratory. To estimate the sample size we powered the study to detect a decrease in FC of 400 mg/l. We assumed that the standard deviation of FC in this group of patients would be 400 mg/l^38^. We calculated that with an 80% power and α = 0·05 (two-sided), 16 patients were needed in each arm of the study.

### Ethical statement

The study was approved by the regional ethics committee (The Regional Committees on Health Research Ethics for Southern Denmark, jr. no.: S-20140133) and by the local Danish Data Protection Agency (number 18/22513). It was conducted in accordance with the Helsinki II declaration.

The animal experiments were conducted in accordance with the Danish law for animal research (Act No. 1306 of 23/11/2007, Danish Ministry of Justice) and Guide for the Care and Use of Laboratory Animals by the US National Institute of Health (NIH Publication No. 85–23, revised 1996).

The study was registered at the US Institute of Health (Clinicaltrial.gov) number NCT02445365 on 15/05/2015. All patients gave written informed consent before inclusion in the study and were given an economic compensation of 1,500 Danish kroner to cover lost income, transportation costs, pain and suffering.

### Statistics

Continuous variables are reported as median and interquartile range (IQR), categorical variables are reported as numbers and percentage. Non-parametric tests were used. For categorical comparison between 2 groups Fischer’s exact test was used. For comparison between more than 2 groups we used Kruskal-Wallis one-way analysis of variance. In case the Kruskal-Wallis one-way analysis of variance showed significant difference we applied pairwise Mann-Whitney U test with Bonferroni correction.

We used linear regression to estimate treatment effect for continuous and categorical outcomes with more than 4 categories. For categorical outcome measures with 4 or less categories we estimated treatment effect by ordinal proportion regression and for binary outcomes we used logistic regression. All regressions were adjusted for baseline (day 1) value of the current variable.

Level of significance was 0.05 except in evaluation of the cytokines where level of significance was 0.01 due to multiple testing.

Sequenced reads from the RNA-seq were normalized to reads per kilobase per million (RPKM), and differentially expressed genes (DEGs) were identified using the DESeq. 2 package^39^ in R 3.2.4. We defined DEGs as genes with log2 fold-change >1.0 and false discovery rate (FDR) < 0.05 to further describe and analyze the transcriptomic differences between the UC patients and the HC. PCA was performed in R 3.2.4 using the prcomp function and visualized using the ggplot2 package^40^. GO analysis was performed with GOseq^41^.

The miRNA reads were normalized to spike-in miR-248. We defined significantly differentially expressed miRNAs as miRNA with log2 fold change >1.0 and p-value <0.01 (expressed as -log10(p-value) >2). PCA was performed as described above.

In the Langendorff experiment we used one-way analysis of variance (ANOVA) for between-groups comparison of means of infarct size/area-at-risk ratio.

Study data were collected in and managed using REDCap (Research Electronic Data Capture) tool hosted at OPEN (Odense Patient data Explorative Network, Department of Clinical Research, University of Southern Denmark)^42^. We did statistical calculations and graphical presentations in R version 3.2.4.

## Results

**Figure 1:**
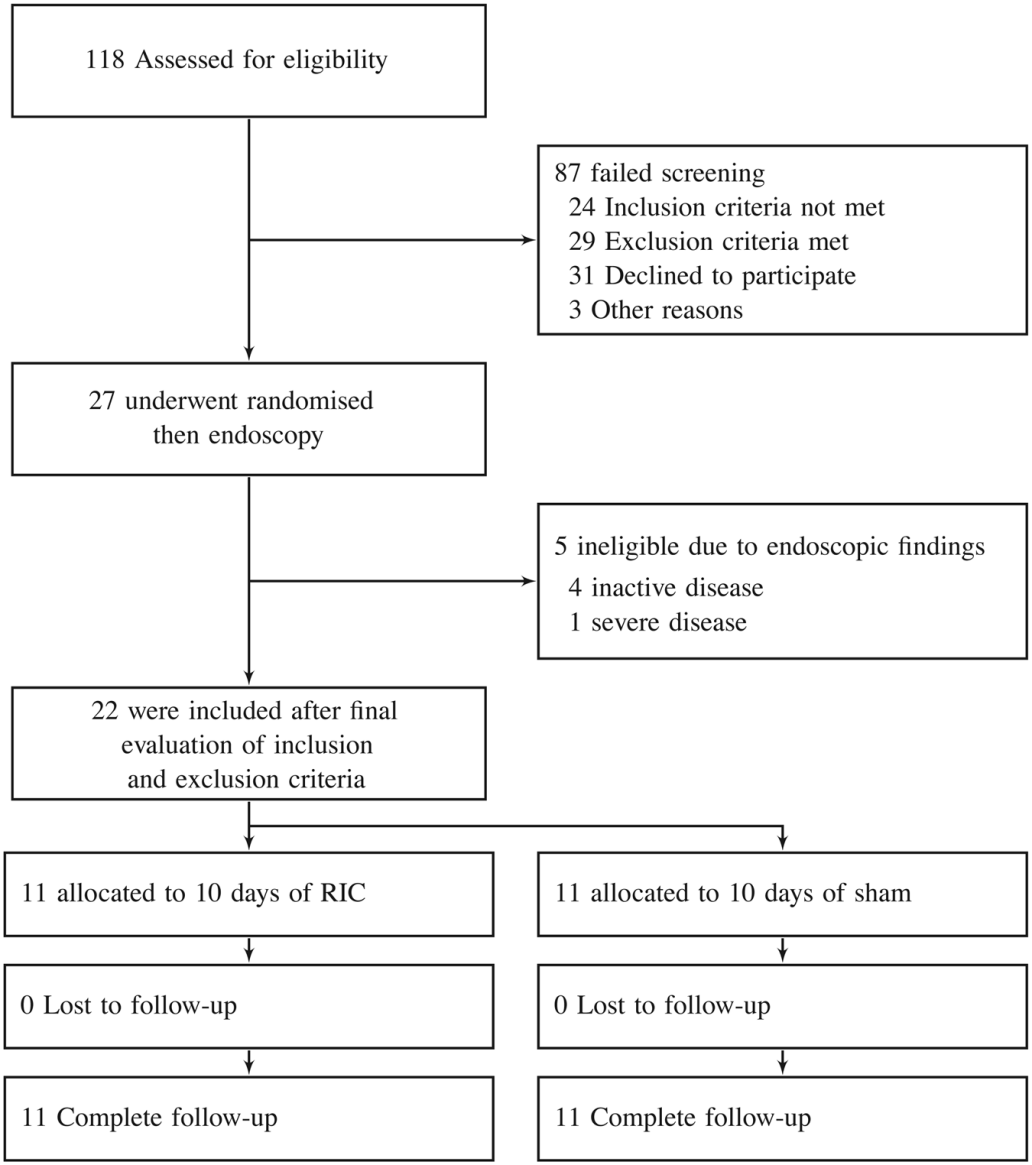
Trial profile.

A total of 118 patients were screened for eligibility during the inclusion period from June 1, 2015 to January 31, 2018. Figure 1 shows trial profile. A total of 87 were excluded before endoscopy, four of 27 randomized patients did not fulfill inclusion criteria after endoscopy. One of the 27 randomized patients was considered too ill based on blood samples taken prior to randomization. Twenty-two patients received the intended intervention and are used in the intension to treat analyses. There were no dropouts during the study period.

Table 1 shows patients’ baseline demographics and clinical characteristics. Significantly more males than females were randomized to RIC compared with sham. Furthermore, there were significantly less patients with no alcohol use in the sham group compared to the RIC group. Otherwise the two study groups were well matched.

**Table 1:** Baseline demographic, clinical and biochemical characteristics of 22 patients with UC.

Characteristic	RIC	Sham
	n = 11	n = 11
Age [years]	41 [36–64]	41 [24–50]
Sex [female]	2 [18%]	9 [82%]
Weight [kg]	78.2 [70.8–91.2]	70.0 [63.9–88.7]
BMI [kg/cm^2^]	25.4 [23.9–28.6]	26.3 [22.6–28.4]
Disease duration [years]	13.2 [8.3–14.9]	5.1 [2.2–12.3]
Smoking status		
Current	1 [9]	0[0]
Former	7 [64]	7 [64]
Never	3 [27]	4 [36]
Alcohol consumption		
≥7/14 alcohol units per week	1 [9]	2 [18]
<7/14 alcohol units per week	10 [91]	5 [45]
No alcohol	0[0]	4 [36]
Disease extent at endoscopy		
Proctitis	4 [36]	6 [55]
Left-sided UC	5 [45]	4 [36]
Pancolitis	2 [18]	1 [9]
Montréal classification		
E1 - Ulcerative proctitis	3 [27]	4 [36]
E2 - Left sided UC [distal UC]	7 [64]	6 [55]
E3 - Extensive UC [pancolitis]	1 [9]	1 [9]
Medication		
No treatment	4 [36]	2 [18]
Rectal 5-ASA	2 [18]	4 [36]
Oral 5-ASA	6 [55]	8 [73]
Immunosuppressants	1 [9]	1 [9]
Anti-TNF	0	0
FC	1220 [523–1808]	1391 [879–4139]
P-CRP	1.95 [1.8–8.3]	1.9 [1.6–6.1]
Mayo Score	7 [6–8]	8 [7–9]
Endoscopic Mayo Score	2 [2–2.5]	2 [2–2.5]
Days of flare prior to inclusion	78 [22.5–150]	41 [22–93]
Days of RIC/Sham	10 [9,10]	10.5 [10,11]

Continuous variables are described as median and IQR, categorical variables are described as frequencies and percentages.

UC, ulcerative colitis; BMI, body mass index; 5-ASA, 5-aminosalicylic acid; Anti-TNF, antitumor necrosis factor inhibitor; FC, Fecal calprotectin; p-CRP, plasma C-reactive protein; RIC, remote ischemic conditioning.

**Figure 2:**
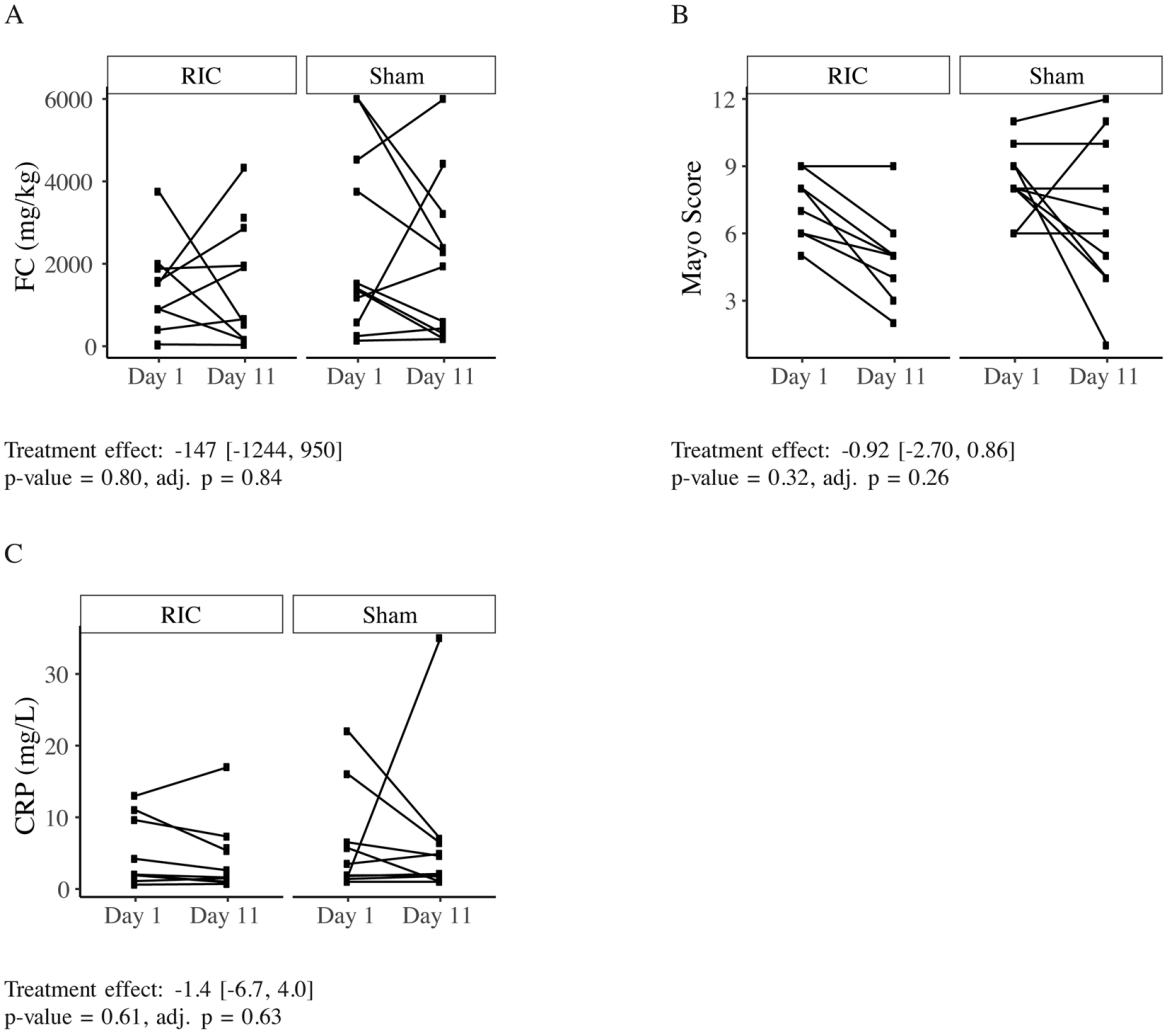
Slope diagrams showing absolute levels of FC, Mayo Score and p-CRP at day 1 and day 11. The treatment effect and 95% CI of each of the variables calculated by linear regression are reported, as well as the p-value and the sex-and age-adjusted p-value. (**A**) FC; (**B**) Mayo Score; (**C**) P-CRP.

The study did not meet its primary endpoint. The treatment effect on the FC was −147 mg/kg (95% CI [−1244, 950]) and was not significant (p = 0.80) (Fig. 2A). There were no significant changes in the secondary outcome including Mayo score, endoscopic Mayo score, Nancy histological index, amount of neutrophils in lamina propria assessed by Geboes histological index Grade 2b, amount of neutrophils in the epithelium assessed by Geboes histological index Grade 3, p-CRP and number of patients achieving remission (Fig. 2B,C, Table 2). When we adjusted the regression models for sex and age at day 1 the treatment effect remained non-significant (Fig. 2A–C, Table 2).

**Table 2:** Change from day 1 at day 11 expressed in median [interquartile range].

	RIC [IQR]	Sham [IQR]	Odd ratio [95%CI]	p-value	Adj. p
Endoscopic Mayo Score	0 [−1, 0]	0 [−1, 0]	0.96 [0.28, 3.33]	0.95	0.68
Nancy Index	0 [0, 0]	0 [−1, 0]	1.15 [0.38, 3.51]	0.80	0.72
Neutrophils in the lamina propria	0 [−1, 0]	0 [0, 0]	0.76 [0.21, 2.69]	0.67	0.17
Neutrophils in the epithelium	0 [−1, 0]	0 [-1, 0]	0.89 [0.27, 2.95]	0.85	0.32
Number of patients achieving remission [%]	1 [9]	1 [10]	0.93 [0.11, 7.29]	0.94	0.99

Treatment effect expressed as odd ratio [95% confidence interval], the associated p-value and p-value adjusted for age and sex.

At Day 30 we assessed basic clinical outcomes to evaluate any long-term clinical effect of RIC. There were no significant differences in FC, partial Mayo score, p-CRP and number of patients achieving clinical remission between the two groups, and when adjusted for sex and age at day 1 the treatment effect remained non-significant (data not shown).

All patients reported daily use of the RIC or sham cuff as assigned at baseline except one patient that did not use the RIC cuff at day 8.

Seven (64%) of the 11 patients assigned to RIC experienced one or more days with minor and momentary side effects in relation to the use of the RIC cuff.

### Cytokines

Seventeen of the 27 assays produced valid measurements including IL-6, IL-10, IL-12/IL-23p40, IL-18, IL-22, IL-27, MMP-1, MMP-2, MMP-3, MMP-7, MMP-8, MMP-9, MMP-10, TIMP-1, ICAM-1, IFN-γ and TNF-α. At day 1, the levels of these cytokines were similar in the two groups (data not shown). We did not observe statistically significant treatment effect on expression levels of any of the cytokine plasma levels neither at day 11 nor at day 1 15 minutes post RIC/sham (data not shown). The concentrations of IL-1β, IL-2, IL-4, IL-12p70, IL-13, IL-17a, IL-17f, IL-21, IL-23 and IL-33 were below detection limits.

### mRNA

**Figure 3:**
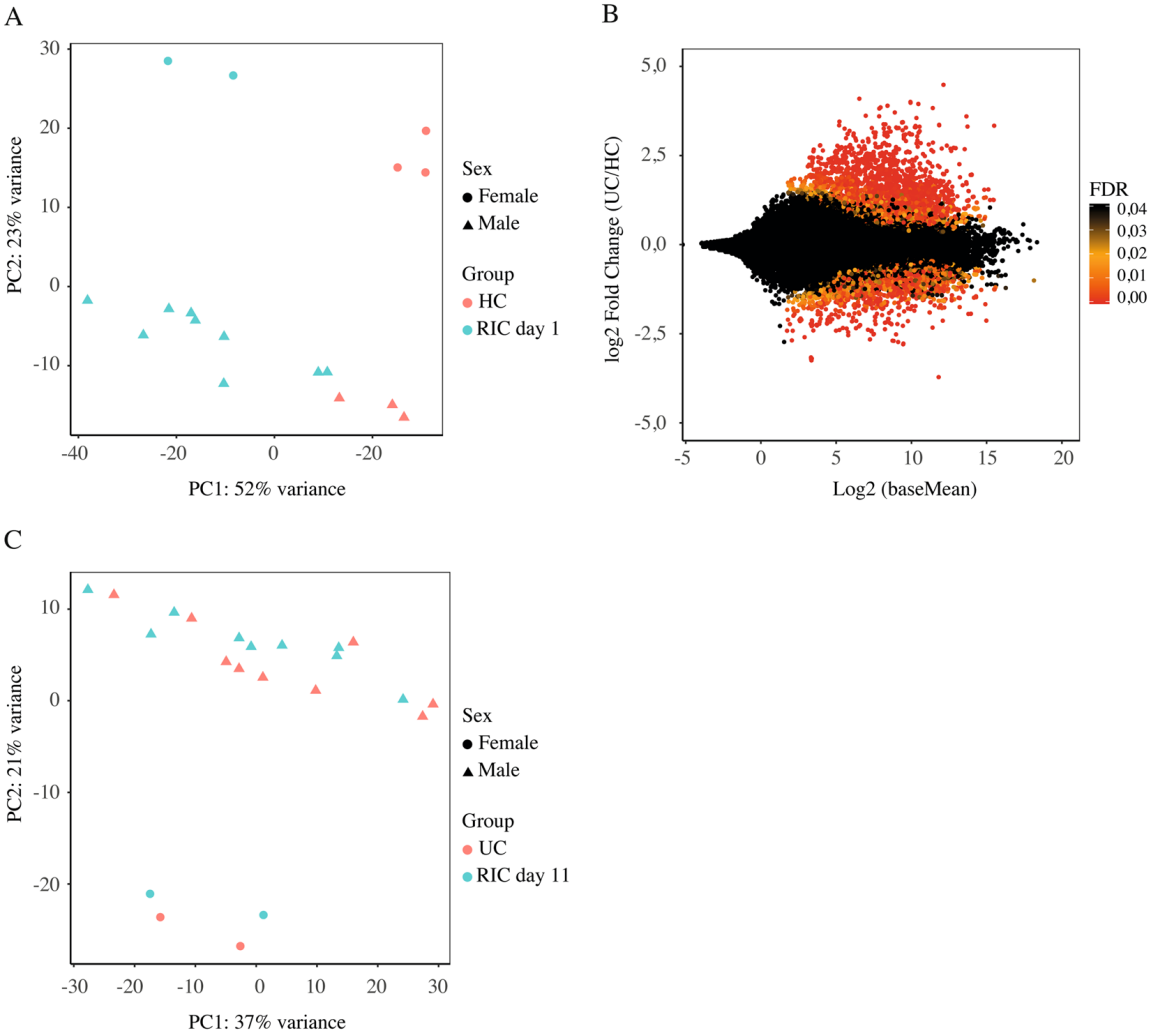
(**A**) PCA plot calculated from mRNA read counts in rectal biopsies of UC (RIC day 1) and HC. (**B**) MA plot showing the relationship between the mRNA expression change between UC and HC (y-axis), the average expression of the genes (x-axis) and the statistically estimated significant level (color legend). (**C**) PCA plot calculated from mRNA read counts in rectal biopsies of RIC day 1 and RIC day 11.

**Figure 4:**
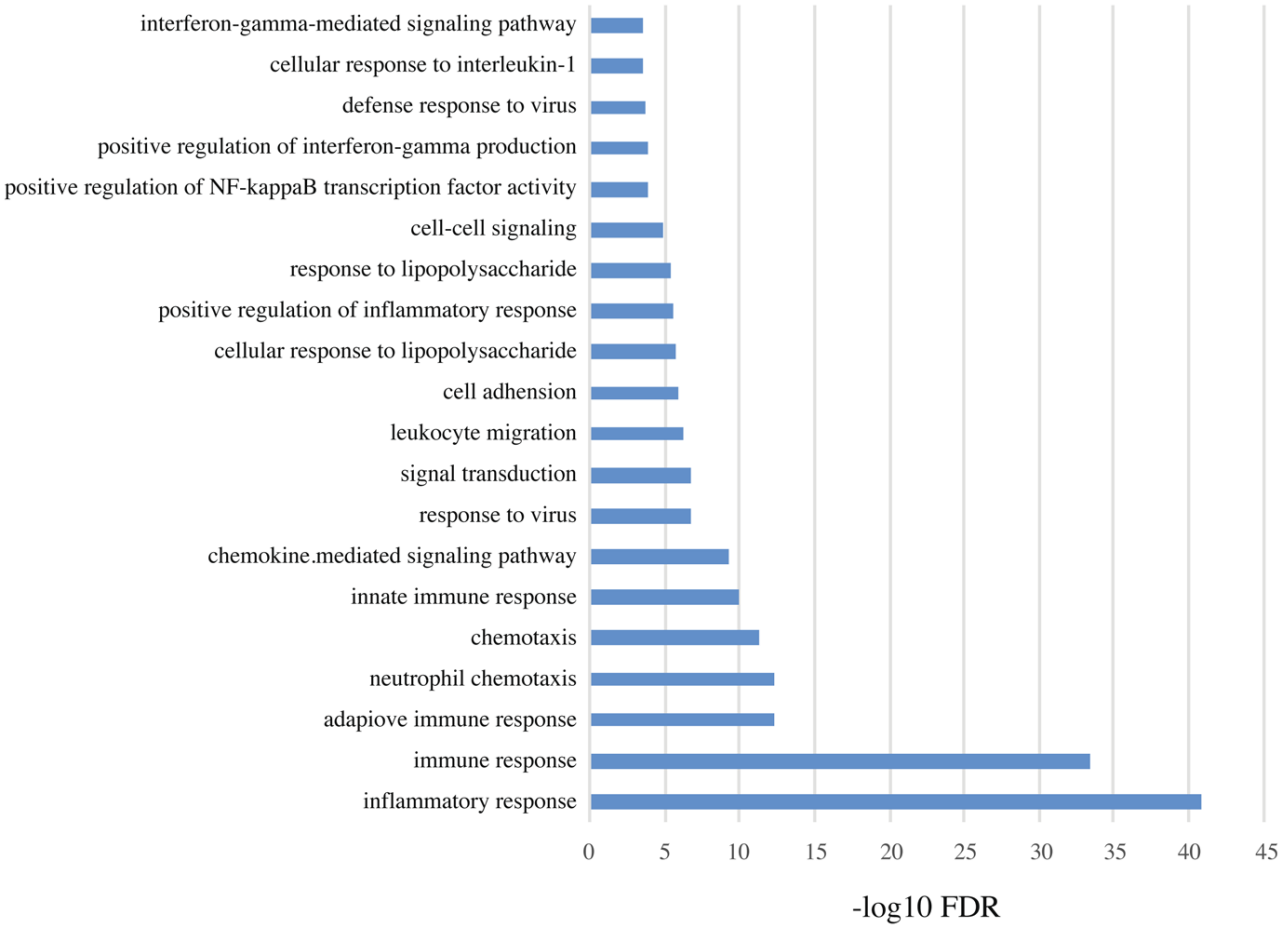
The twenty most regulated GO term pathways. X-axis is log10 with Bonferroni correction.

The RNA-seq quantified expression of approximately 58,000 annotated transcripts. Principal component analysis (PCA) plot showed a clear separation of patients with active UC (RIC group day 1 and sham group day 1) and HC along the first principal component with a 52% explained variance. The second principle component explained 23% of the variance and it likely associated with the sex of the participants (Fig. 3A). More than 3,600 DEGs were detected in the UC cohort compared to HC controls (Fig. 3B). Gene ontology (GO) term enrichment analysis suggested that the differentially regulated mRNAs are primarily involved in inflammatory and immune pathways. The 20 most significantly regulated pathways are listed in Fig. 4.

Subsequently, we compared variability between the mRNA profile in the RIC group at day 1 and day 11 (Fig. 3C) and variability between the RIC group day 11 and the sham group at day 11 (not shown) to evaluate the effect of RIC on the gene expression. This analysis revealed a clear clustering based on sex and little variability related to the RIC and sham intervention. This was furthermore confirmed in a sex-matched PCA comparing the 9 males in the RIC group at day 1 and day 11, which showed no clear clustering of data (data not shown).

### miRNA

Of the 798 miRNAs measured 87 had 10% or more of the samples above background count. These were selected for further analyses. Two of the 68 samples (one from the RIC group day 1 before intervention and one from the RIC group day 11) used for miRNA profiling were hemolyzed and therefore left out of the following analyses.

**Figure 5:**
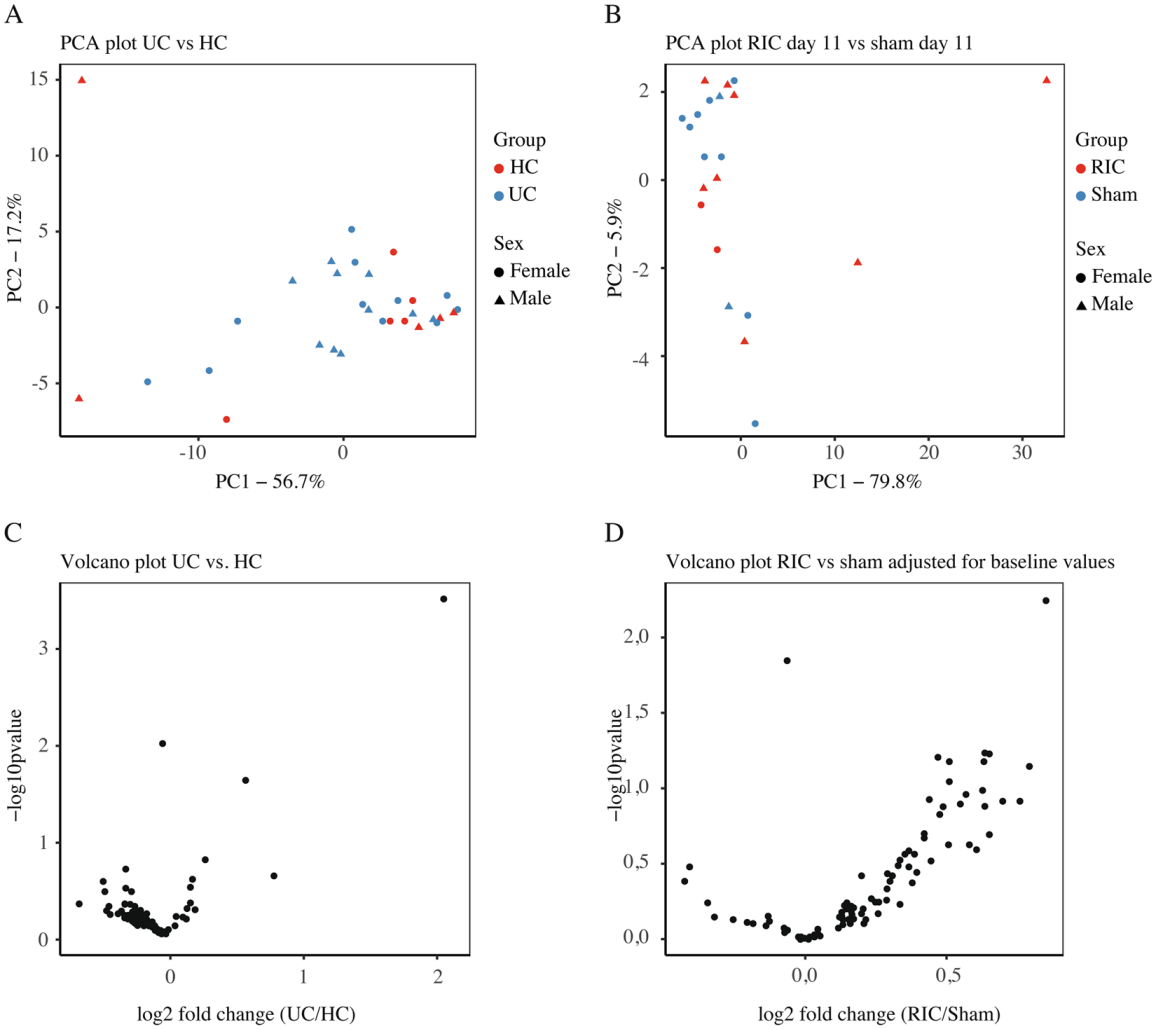
(**A**) PCA plot calculated from miRNA read counts in plasma samples from UC patients (RIC and sham groups day 1 before intervention) and HC. (**B**) PCA plot calculated from miRNA read counts in plasma samples from the RIC group day 11 and the sham group day 11. (**C**) Volcano plot showing fold change (x-axis) and the statistical significance (y-axis) of each miRNA comparing the UC group (RIC and sham groups day 1 before intervention) and HCs. (**D**) Volcano plot showing fold change (x-axis) and the statistical significance (y-axis) of each miRNA comparing the RIC group at day 11 and the sham group at day 11 corrected for baseline values of read counts.

PCA plot of miRNA data from 21 UC patients with active disease (RIC day 1 and sham day 1) and the 10 HC did not show any clustering of data relating to these groups (Fig. 5A). Subsequently, we compared variability between the miRNA profile in the RIC group at day 11 and the sham group at day 11 (Fig. 5B) to evaluate the effect of RIC on the miRNA expression. There were no clear clustering based on the intervention group or sex. The same was seen when the RIC group and the sham group were compared before the first intervention and 15 minutes after the first intervention (data not shown).

Subsequently, we analyzed differential expression of miRNAs by comparing the RIC group at day 1 and the sham group at day 1, collectively denoted UC, to HC (Fig. 5C), as well as RIC day 11 and sham day 11 adjusted for day 1 miRNA expression (Fig. 5D), and RIC day 1 15 minutes after first intervention and sham day 1 15 minutes after first intervention adjusted for day 1 miRNA expression (data not shown). miR-1246 was the only one of the 87 miRNAs that was statistically significant differentially expressed in the UC group compared to the HC group. None of the 87 miRNAs were statistically significant differentially expressed in the RIC day-11 group compared to the sham day-11 group.

miR-144 was generally highly expressed in all samples. Comparing RIC day 11 to sham day 11 after adjusting for baseline values, levels of miR-144 expression show a log2(fold change) = 0.51 and p-value = 0.23 indicating that miR-144 is not differentially expressed in the RIC group compared to the sham group after 10 days of daily RIC/sham intervention in patients with UC.

### The Langendorff heart model

**Figure 6:**
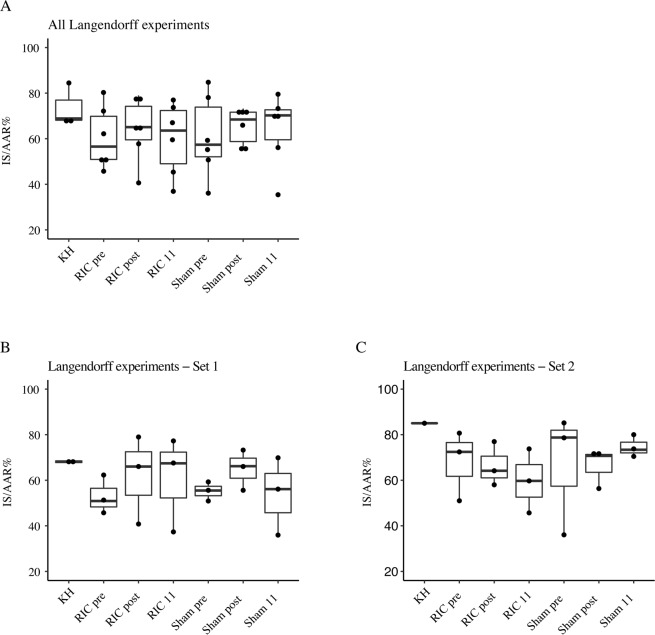
Box-dot plot showing the IS/AAR (%) in the RIC and the sham groups at the different time points. (**A**) All Langendorff experiments (6 from the RIC group, 6 from the sham group, 3 KH), (**B**) Set 1 of Langendorff experiments (3 from the RIC group, 3 from the sham group, 2 KH). (**C**) Set 2 of Langendorff experiments (3 from the RIC group, 3 from the sham group, 1 KH). Pre, day 1 before the first intervention; post, 15 minutes post the first intervention; 11, after 10 days of daily interventions.

Figure 6 shows the infarct size/area-at-risk ratio (IS/AAR) from all Langendorff analyses as well as the IS/AAR divided into the 2 sets. There was no difference in infarct IS/AAR between the pre- and post-RIC groups and no difference between the RIC group and the sham group.

Isolated rat hearts perfused with plasma from the RIC-treated patients did not improve post-ischemic cardiac performance measured by peak left ventricular developed pressure calculated as the difference between the systolic and the end-diastolic LV pressures, cardiac flow (CF) in ml/min or rate pressure product (RPP) (data not shown).

Plasma was drawn before and approximately 30 minutes after RIC in 3 healthy males. RIC reduced the mean IS/AAR% from 80.06 to 63.04 (p-value 0.034) in the healthy controls confirming the ability the Langendorff setup to detect the cardioprotective effect of RIC.

## Discussion

To our knowledge, this is the first study of RIC in active UC patients and in an inflammatory condition in humans. After 10 days of daily RIC or sham intervention, we did not observe a reduction in FC, clinical symptoms, endoscopic or histological activity scores in the RIC group compared to the sham group. Nor did we detect a reduction in surrogate serological markers of inflammation or a change in circulation cytokines as response to RIC. We did not detect an increased number of patients achieving remission in the RIC group compared to the sham group. RIC was well tolerated and adherence to treatment was high. We could not document a significant change in the expression of the mRNA or miRNA transcripts, overall or individually, in relation to the RIC intervention. Finally, we did not detect a reduction in infarct size in the rat hearts perfused with plasma from the RIC group indicating that the organ protection is not induced in the UC patients with active inflammation.

To evaluate the effects of RIC in UC patients, we used a randomized clinical trial design. Hereby, we compared RIC with sham directly and minimized the risk of bias and confounding. Active UC is a good model for *in vivo* chronic inflammation, as the inflamed tissue is accessible by endoscopy and can be studied macroscopically as well as histologically. In the study we evaluated a wide range of outcomes including standard clinical and biochemical methods of measuring disease activity and inflammation in UC patients, as well as newer methods measuring alteration in inflammatory cytokines and gene expression, which directly reflect the pathophysiology of UC. We were not able to directly or indirectly observe effects or any consequences of these effects of RIC on the primary or secondary outcomes. This indicates that RIC does not have an anti-inflammatory effect in patients with active UC.

Cytokines like IL-1β, IL-6, IL-10 and TNF-α and MMPs are associated to disease activity in UC^4,43^, as well as to cardiac or intestinal I/R injury^13,44,45^, and to be attenuated by RIC as stated earlier. This attenuation in cytokine levels was not seen in the current study. Some of these cytokines have been evaluated as potential signal molecule in RIC, however, out of 25 molecules measured only IL-1α changed sufficiently to be a potential marker or mediator of RIC^46^. Cytokines involved in UC have also been evaluated as potential as markers of disease activity and the results inconsistently^47^. Cytokines are subject to multilayer regulation and the changes might be more pronounced in tissue than in the circulation.

RIC has been shown to reduce leucocyte adhesion to the endothelium in HC^16–18^. We assessed the biopsies by Geboes histological score grade 2b, assessing neutrophils lamina propria, and grade 3, assessing neutrophils in the epithelium^30^, to study if there was a clinical relevant decrease in neutrophil infiltration. However, we could not document this. The two scores have not been validated as separate measurements and might be too insensitive to detect a potential small decline in neutrophils granulocytes in the mucosa in this study.

We used RNA-seq and NanoString nCounter to measure mRNAs and miRNAs, respectively. RNA-seq uses deep-sequencing technology and is able to read the complete set of annotated transcripts in the selected tissue sample^48^, whereas the NanoString nCounter using hybridization is tailored to measure 800 miRNA^49^. Both methods yield broad profiles, and the methods are suitable for comparing relative abundance of RNAs^49^. Changes in expression of mRNAs or miRNAs are not validated as markers or methods to evaluate treatment effect in patients with active UC. However, transcriptome studies in patients with UC have shown that mRNAs are differentially expressed in patients with active UC, UC in remission and HCs^50,51^. Furthermore, mRNAs significantly change expression profile during a 14-week treatment period in patients with UC^52^. Also miRNA are differentially expressed in UC patients compared to HCs when analyzing mucosal biopsies and peripheral blood^53,54^. Studies of gene regulation in subjects treated with RIC have demonstrated altered gene transcription in the target organ and peripheral blood 15 minutes and 24 hours after remote ischemic preconditioning^55,56^.

We demonstrated an altered gene expression between UC patients and HCs, which relates to up-regulation of the inflammatory and immune response that are likely to be involved in the pathogenesis of UC, as well as differences explained by sex. This is in line with previous findings^50^. Furthermore, the increased expression of miR-1246 in UC patients compared to HCs has also been demonstrated before^57^.

The lack of significant changes in mRNA and miRNA profiles as response to RIC could either indicate that RIC has no effect in patients with active UC, or that the chronic inflammation, for unknown reasons, overshadows the effects of RIC. Also differences in diet restrictions before blood and tissue sampling may influence the result. In relation to RIC, miR-144 has been suggested to be both a biomarker for and a humoral factor involved in RIC-induced cardioprotection^58^, but we could not confirm that.

If the absent effect of RIC is not due to methodological explanations, there are overall two reasons for RIC not affecting disease activity in UC patients: 1) the protective mechanisms are not induced in UC patients with active disease; or 2) the protective mechanisms are induced but do not affect UC. Whittaker and Przyklenk discuss the phenomenon of hyperconditioning indicating that RIC might have a biphasic dose-response curve inducing protection at low doses, which are lost at high doses^59^. Some of the triggers suggested to be immediately released as response to RIC are adenosine^60^ and TNF-α^61^. Adenosine is in general found to be elevated in inflammation^62^ and TNF-α is elevated in UC patients as part of the inflammatory response during disease activity^63^. Hence, some of the triggers of RIC may already be present in the UC patients and have been present for days. Hereby, RIC mechanisms may already be activated in the UC patients. This is a major difference from controlled setting in animal studies where the animals are healthy prior to the intervention and the mediators increase due to conditioning. If mediators in inflammation like transcutaneous electrical nerve stimulation^64^ and capsicum^65^ can induce organ protection or stimulate the protective signal pathway, it is easy to speculate that the dose of such protective triggers might have been crossed prior to RIC in the UC patients with active inflammation. Hence, RIC does attenuate acute inflammatory response in I/R injury and not the chronic inflammatory response in UC.

The effect of RIC is stronger and more evident in animal studies than in human clinical studies. Translation from basic science to clinical use has been challenging, even in the cardiological ischemia/reperfusion-injury setting, where the evidence of protection is strongest. Several small studies have shown clinical effect^66,67^, but the effect has failed to be validated in large-scale, multicenter study until now^68,69^. In general, extrapolation from animal studies to human studies can be difficult. Animal studies often use uniform, young, inbred, otherwise healthy male animals. In contrast, human subjects in clinical trials are heterogeneous in perspective of older age, co-morbidities and medication^70^, which may attenuate the effect of RIC^70,71^. Several conditions like diabetic neuropathy, hypercholesterolemia, age or male sex has been found to disrupt or decrease the protection otherwise induced by RIC^13,72^. In the current study sex and age could play a role in inhibiting or decreasing the effect of RIC since the RIC group is mainly males and they were, although not significantly, older than the females. Furthermore, the current study population is very heterogeneous in regard to difference in disease activity (both degree of inflammation and extent of disease), weight, smoking habits, coffee and alcohol consumption, and hormone levels.

It could also be considered whether the patients in our study with active UC received sufficient RIC. The RIC algorithm of four cycles of alternating five minutes ischemia and five minutes reperfusion used in the current study was the common practice at the time this study was initiated. This was confirmed to be within the limits of the optimal RIC algorithm suggested by Johnsen *et al*. in 2016^73^. The duration of 10 days of RIC was chosen partly because established treatments for active UC are effective within 1–2 weeks. Thus new treatment modalities should be as effective as current therapy. Furthermore, partly due to ethical consideration concerning the duration of withholding known effective treatment to the patients with active UC, especially for patients in the sham group.

We used the Langendorff heart model to explore if the organ protective mechanisms initiated by RIC were induced in the UC patients. We did not see any differences in infarct size between the two groups, which could indicate that the organ protective mechanism is not induced. Moreover, we did not see an improvement in post-ischemic cardiac performance in the hearts perfused by plasma from the RIC group, which further indicates that the cardioprotective mechanism is not induced in this group of UC patients.

The lack of activation of the cardioprotective mechanisms seen in the UC patients is also seen in studies of patients with diabetes^74^. A study showed that activation and protection of RIC in patients with type 2 diabetes mellitus was dampened in the subgroup of patients with neuropathy but not in the subgroup of patients without neuropathy, indicating the need of an intact neuronal pathway^75^. Another study in patients with type 1 diabetes mellitus showed that these patients were more resistant to ischemia and reperfusion than healthy controls, and RIC did not add further cardioprotection to the patients with type 1 diabetes mellitus as it did in the healthy controls. This could indicate that the patients with type 1 diabetes mellitus are already in a conditioned state^76^. The autonomic nervous system is also known to be involved in UC, especially the vagus nerve seem to be involved^25,26^. A study has shown autonomic nerve dysfunction in IBD with vagal nerve dysfunction in UC patients compared to healthy controls^77^, and another study has shown increased inflammation in UC rat models undergoing vagotomy compared to UC rat models with an intact vagus nerve^78^. This could indicate that the cholinergic anti-inflammatory pathway is already activated in UC patients and cannot be further stimulated by RIC. Similar to the type 1 diabetes mellitus patients the UC patients may already be in a conditioned state. Post hoc we compared the mean IS/AAR% from UC patients before the first intervention (60.70 +/− SEM 4.26) to the 3 healthy controls (80.06 +/− SEM 4.23) which showed that the UC patients had a substantially reduced IS/AAR% compared to the healthy controls which substantiates this hypothesis further. However, these numbers should be interpreted with caution.

Anti-inflammatory medications like immunosuppressant and biologicals used in UC could potential interfere with RIC since they partly affect the same signal mechanism. However, only one patient in each intervention group in our study received immunosuppressant and none received biologicals. The most used treatment in our study cohort was 5-ASA, which is less likely to interfere with RIC because it acts locally in the gut.

The study has some limitations. A major limitation is the small sample size and the failure to reach estimated sample size implying the risk of not demonstrating a true difference in the primary outcome between the two groups. The skewed randomization by sex could bias the result, but did not seem to do so when we statistically adjusted for sex in the logistic regression models estimating treatment effect. Complete blinding of the participants was not possible due to the nature of RIC, which is also shown by the high number of participants guessing the assigned intervention group. Ten (90%) of the patients in the RIC group and eight (73%) in the sham group knew by guessing which group they were randomized to. One (9%) in each group did not know which group they were randomized to, and two (18%) in the sham group thought they were randomized to the RIC group. In general, patients had a positive attitude toward the intervention.

In conclusion, this study cannot document that RIC modulates or reduces inflammation in patients with active UC. We did not see attenuation in clinical, endoscopic or histological disease activity scores, we did not see attenuation in serological and fecal biomarkers of inflammation, we did not see a change in mRNA or miRNA expression profile, and we could not confirm the induction of cardioprotection otherwise induced by RIC. Whether UC patients are already conditioned, or UC/inflammation inhibits the protective mechanism of RIC is not revealed by this study, although it is likely that the UC patients, and maybe chronic inflamed patients in general, are in a conditioned state. This should be taken into account in future studies of RIC involving patients with chronic inflammation.

## Data Availability

All data will be made available upon reasonable request.
